# Supplementary material for: Identification of digital clinical decision support systems for supporting diagnosis and triage of patients with shoulder disorders: A scoping review protocol
Source: PLoS One. 2025 Jul 1;20(7):e0327192. doi: 10.1371/journal.pone.0327192 (PMC12212480; doi:10.1371/journal.pone.0327192)
Supplement: S2 Appendix — (DOCX) [file pone.0327192.s002.docx]

S2 Appendix. Search strategy using OVID Medline.

| **#** | **String** | **Type** | **Results** |
| --- | --- | --- | --- |
| 1 | exp artificial intelligence/ or exp clinical decision-making/ or exp clinical decision rules/ or exp critical pathways/ or exp decision making, computer-assisted/ or exp decision support systems, clinical/ or exp decision support techniques/ or exp diagnosis, computer-assisted/ or exp digital health/ or exp expert systems/ or exp image processing, computer-assisted/ or exp image interpretation, computer-assisted/ or exp medical order entry systems/ or exp point-of-care systems/ or exp therapy, computer-assisted/ | Advanced | 12050322 |
| 2 | decision support*.mp | Advanced | 52362 |
| 3 | (cdss OR cpoe OR dss OR edss OR ebds OR hdss OR icds OR idss OR mdss OR pdss OR poeds OR tdss).mp. | Advanced | 37272 |
| 4 | (clinical ADJ3 system*).mp. | Advanced | 57832 |
| 5 | (artificial intelligen* OR clinical assistan* OR AI clinical assistan* OR computer-assisted diagnosis system* OR diagnostic support system* OR disease management system* OR healthcare cognitive computing system* OR knowledge-based clinical system* OR medical logic module* OR medical recommendation system* OR smart clinical assistan*).mp. | Advanced | 96036 |
| 6 | (decision ADJ3 (aid$ or alert OR algorithm$ OR application$ OR assistan$ OR cognitiv$ OR comput$ OR guid$ OR intelligen$ OR knowledge management OR pathway$ OR predict$ OR program$ OR reason$ OR recommend$ OR resourc$ OR rule$ OR software OR system$ OR technolo$ OR tool$ OR workflow$)).mp. | Advanced | 78604 |
| *7* | *1 OR 2 OR 3 OR 4 OR 5 OR 6* | Advanced | *1366533* |
| 8 | exp acromioclavicular joint/ OR exp brachial plexus/ OR exp brachial plexus neuropathies/ OR exp rotator cuff/ OR exp rotator cuff Injuries/ OR exp scapula/ OR exp shoulder/ OR exp shoulder dislocation/ OR exp shoulder dystocia/ OR exp shoulder fractures/ OR exp shoulder impingement syndrome/ OR exp shoulder injuries/ OR exp shoulder joint/ OR exp shoulder pain/ OR exp sternoclavicular joint/ | Advanced | 90611 |
| 9 | (shoulder$ ADJ3 (abduct$ OR adduct$ OR bursitis OR capsulitis OR arthrit$ OR contracture$ OR contusion$ OR coracoacrom$ OR degenerat$ OR diseas$ OR dislocat$ OR frozen OR glenohumer$ OR glenoid$ OR humeroscapular$ OR hyoerexten$ OR hypermob$ OR impingement$ OR infect$ OR injur$ OR instab$ OR osetolysis OR pain OR scapul$ OR separation OR sprain OR strain OR sublux$ OR suprascapul$ OR swell$ OR tear$ OR tendin$ OR unstab$ OR wound$)).mp. | Advanced | 40714 |
| 10 | *8 OR 9* | Advanced | *102941* |
| 11 | exp community health centers/ OR exp community health nursing/ OR exp community health services/ OR exp community health workers/ OR exp delivery of health care/ OR exp family practice/ OR exp general practice/ OR exp general practitioners/ OR exp health personnel/ OR exp physicians/ OR exp physicians, family/ OR exp physicians, primary care/ OR exp preventive health services/ OR exp primary health care/ | Advanced | 2658728 |
| 12 | (community health OR comprehensive care continuing care OR continuity of care OR family doctor$ OR family medicine OR family physician$ OR family practic$ OR first-contact care OR frontline healthcare OR health-care OR healthcare OR health care OR health promotion OR health clinic$ OR health screening OR health visit$ OR integrated care OR physician assistant$ OR point-of-care OR preventive care OR primary care OR primary care physician$ OR primary care provider$ OR primary health care OR primary healthcare OR primary healthcare services).mp. | Advanced | 1680406 |
| 13 | *11 OR 12* | Advanced | *3384072* |
| 14 | 7 AND 10 AND 13 | Advanced | 325 |
| 15 | limit 14 to english language | Limiter | 305 |
| 16 | limit 15 to humans | Limiter | 301 |
| 17 | limit 16 to (clinical study or clinical trial, all or clinical trial or comparative study or controlled clinical trial or "corrected and republished article" or evaluation study or journal article or meta analysis or multicenter study or network meta-analysis or observational study or pragmatic clinical trial or preprint or published erratum or randomized controlled trial or "scoping review" or "systematic review" or validation study) | Limiter | 294 |
|  | Note This search was run and documented on 10 April 2025, using Ovid MEDLINE ALL 1946 to April 09, 2025. |  |  |
